# The value of intraoperative neuromonitoring combined with high-definition endoscopy in the operation of brachial plexus schwannoma

**DOI:** 10.3389/fsurg.2026.1734352

**Published:** 2026-04-08

**Authors:** Yuxuan Xing, Junguo Wang, Xiaohui Shen, Dengbin Ma, Jiayi Li, Xiao Wu, Handong Wang, Yajun Gu, Xiaoyun Qian

**Affiliations:** 1 Jiangsu Provincial Key Medical Discipline (Laboratory), Department of Otolaryngology-Head and Neck Surgery, Nanjing Drum Tower Hospital, Clinical College of Nanjing Medical University, Nanjing, Jiangsu, China; 2 Jiangsu Provincial Key Medical Discipline (Laboratory), Department of Otolaryngology-Head and Neck Surgery, Nanjing Drum Tower Hospital, The Affiliated Hospital of Nanjing University Medical School, Nanjing, Jiangsu, China; 3 Research Institute of Otolaryngology, Nanjing, Jiangsu, China; 4 Department of Anesthesiology, Nanjing Drum Tower Hospital, The Affiliated Hospital of Nanjing University Medical School, Nanjing, Jiangsu, China

**Keywords:** brachial plexus, endoscopic system, intraoperative nerve monitoring, motor function, Schwannoma

## Abstract

**Background:**

To investigate the value of intraoperative neuromonitoring (IONM) combined with high-definition endoscopy in surgical treatment of brachial plexus schwannoma.

**Methods:**

A retrospective analysis was conducted on twenty patients diagnosed with brachial plexus schwannoma from January 2020 to December 2024. All cases were treated surgically with IONM combined with high-definition endoscopy. Intraoperative and postoperative nerve function were assessed to evaluate the value of this combined approach during surgery.

**Results:**

All twenty patients underwent complete intracapsular tumor resection. Two patients developed numbness in the fingers, and one patient developed numbness in the shoulder. Postoperative motor function was unaffected in all patients. No tumor recurrence was observed during a follow-up period from one to four years.

**Conclusion:**

IONM combined with high-definition endoscopy helps to better identify nerve trajectories, plan tumor envelope incision pathways, detect early nerve injuries and assess the prognosis of nerve conduction function. This procedure also contributes to improving patient's quality of life.

## 1 Introduction

Schwannoma is a type of benign nerve tumor composed of Schwann cells surrounding peripheral, cranial, and autonomic nerves (1). It usually occurs in the limbs and the head and neck region, with a slightly higher prevalence in female patients between 30 and 60 years of age (2). Primary tumors of the brachial plexus account for 5% of all upper limb tumors and Schwannoma is the most common type (3). In the region of the brachial plexus from the neck to the armpit, the presence of isolated, slow-growing, round or oval mass with clear boundaries against the surrounding tissues should lead to the consideration of brachial plexus schwannoma. The tumor location can be determined by computed tomography (CT), three-dimensional short-term inversion recovery sampling perfection with application-optimized contrast using different flip angle evolution (3D-STIR-SPACE), and ultrasonography (4) preoperatively. However, a definitive diagnosis can only be obtained through histological analysis. Surgical resection is the primary treatment option for patients with brachial plexus schwannoma and complete tumor removal to reduce recurrence rates while minimizing intraoperative nerve function damage is the key to better outcomes (5). Intraoperative neuromonitoring (IONM) evaluates the functional integrity of nerves in real time during surgery by applying electrical stimulation and continuous recording (6). This allows the assessment of the continuity of nerves and the integrity of nerve–muscle conduction function; high-definition endoscopy has the advantages of strong visual clarity and high precision. Surgeons can obtain clear, real-time images through the system, while simultaneously using proper instrumentation to perform fine operations of the lesion (7).

This study retrospectively analyzed the data of twenty patients with brachial plexus schwannoma. All patients were treated with IONM combined with high-definition endoscopy in the Department of Otorhinolaryngology-Head and Neck Surgery at Nanjing University Medical School Affiliated Drum Tower Hospital from January 2020 to December 2024. By examining the intraoperative and postoperative nerve function of these patients, the study aims to explore the application value of IONM combined with high-definition endoscopy in the surgical process.

## 2 Materials and methods

### 2.1 Patients

Twenty patients with brachial plexus schwannoma from January 2020 to December 2024 were included in this retrospective study. All patients underwent IONM combined with high-definition endoscopy. Information such as age, sex, preoperative symptoms, tumor size and location, CT and 3D-STIR-SPACE sequence, intraoperative and postoperative neurological function levels were recorded and analyzed.

### 2.2 Monitoring of motor nerve function

**Figure 1:**
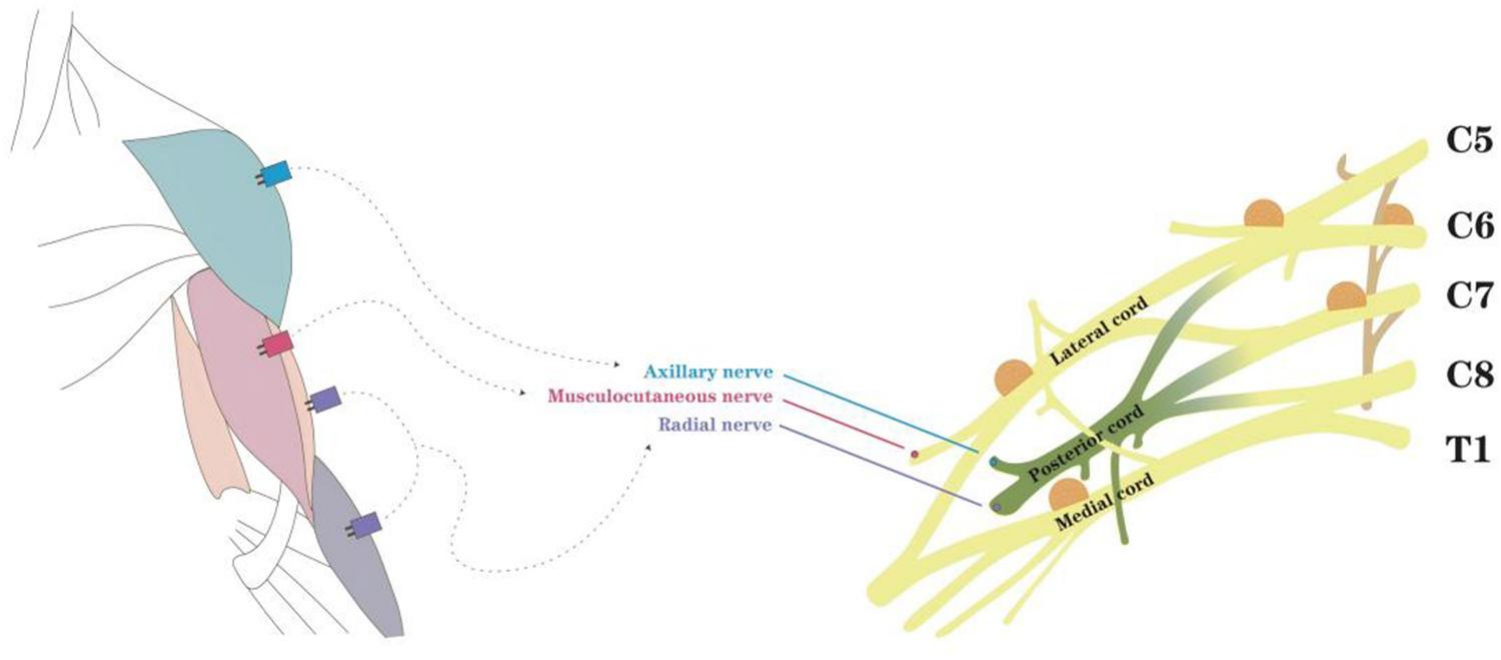
Schematic of intraoperative muscle monitoring.

Motor nerve function was monitored using the Nicolet EDX system (San Carlos, USA). Paired subdermal needles were inserted into the target muscles (Figure 1). During surgery for brachial plexus tumors, intraoperative neuromonitoring was performed using motor evoked potentials (mEPs), somatosensory evoked potentials (ssEPs), and direct nerve stimulation. After stimulation of the peripheral nerve at the upper extremity, ssEP was recorded from the transcranial electrode. The stimulus was given automatically every 15 min. mEPs were evoked by using transcranial electrical stimulation with multi-pulse or train stimulation technique, and recorded fromthe target muscle. Direct nerve stimulation was used to make sure when the structure was uncertain whether it was nerve or not, by bipolar stimulation forceps intraoperatively. This integrated approach aided in intraoperative decision-making regarding the preservation or sacrifice of suspicious neural structures.

### 2.3 High-definition endoscopy

The high-definition endoscopic system was mainly purchased from XION (Berlin, Germany) to provide visualization. Open surgical instruments were used.

### 2.4 Surgical procedure

**Figure 2:**
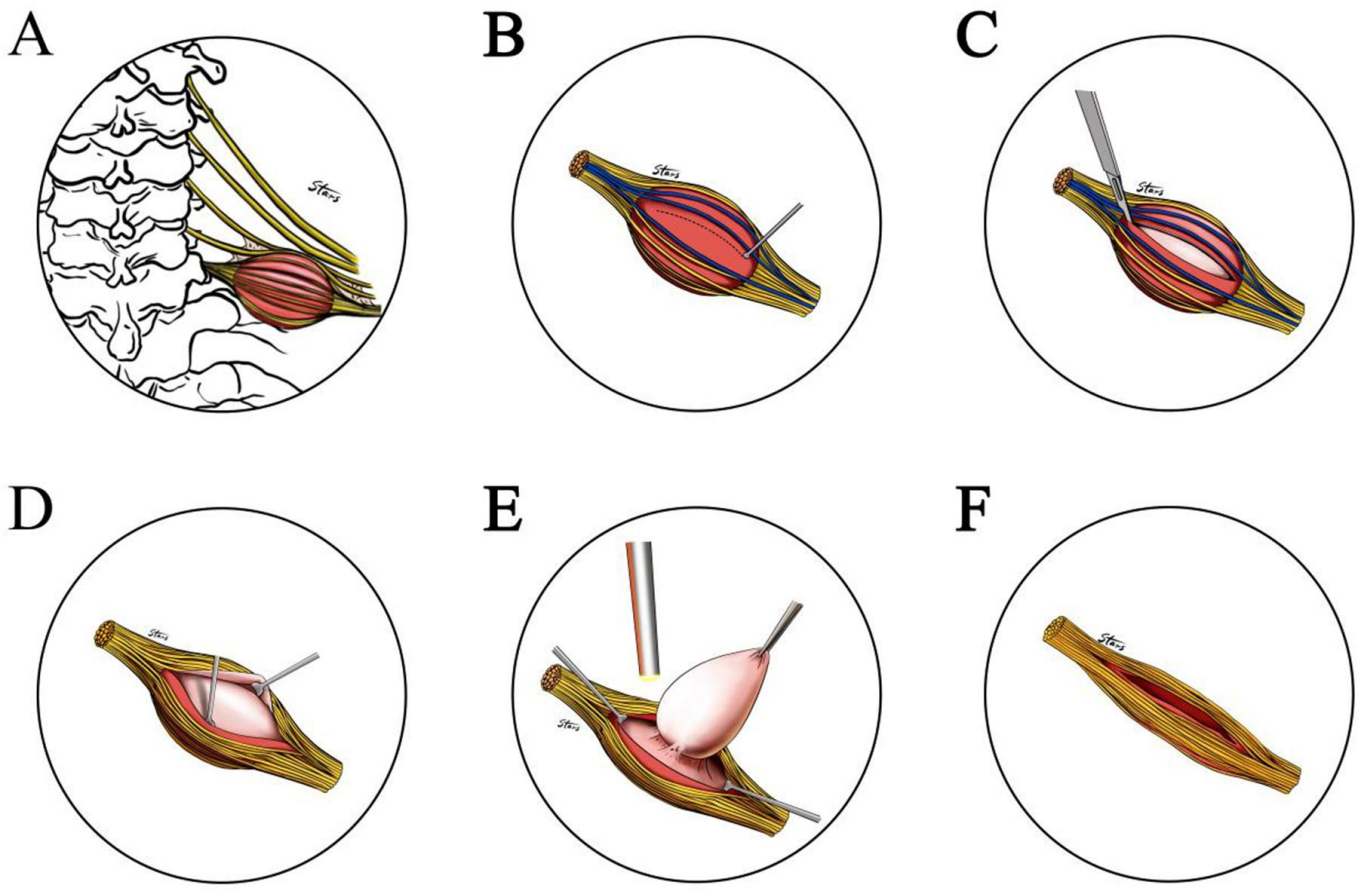
Schematic of brachial plexus schwannoma resection. **(A)** Schwannoma was observed on the C6 branch of the brachial plexus. **(B)** Using the stimulation probe, the neural course was identified (yellow) and marked (blue solid line), and the incision route bypassing the nerve was planned (blue dotted line). **(C)** The capsule was cut along the planned route. **(D)** An extractor was used to carefully peel open the capsule and expose the tumor. **(E)** The tumor was retracted with non-invasive forceps. The high-definition endoscope was used to closely observe the tail of the tumor, ensuring the integrity nerve bundles on the inner and outer side of the capsule. **(F)** The capsule was restored to its original position.

**Figure 3:**
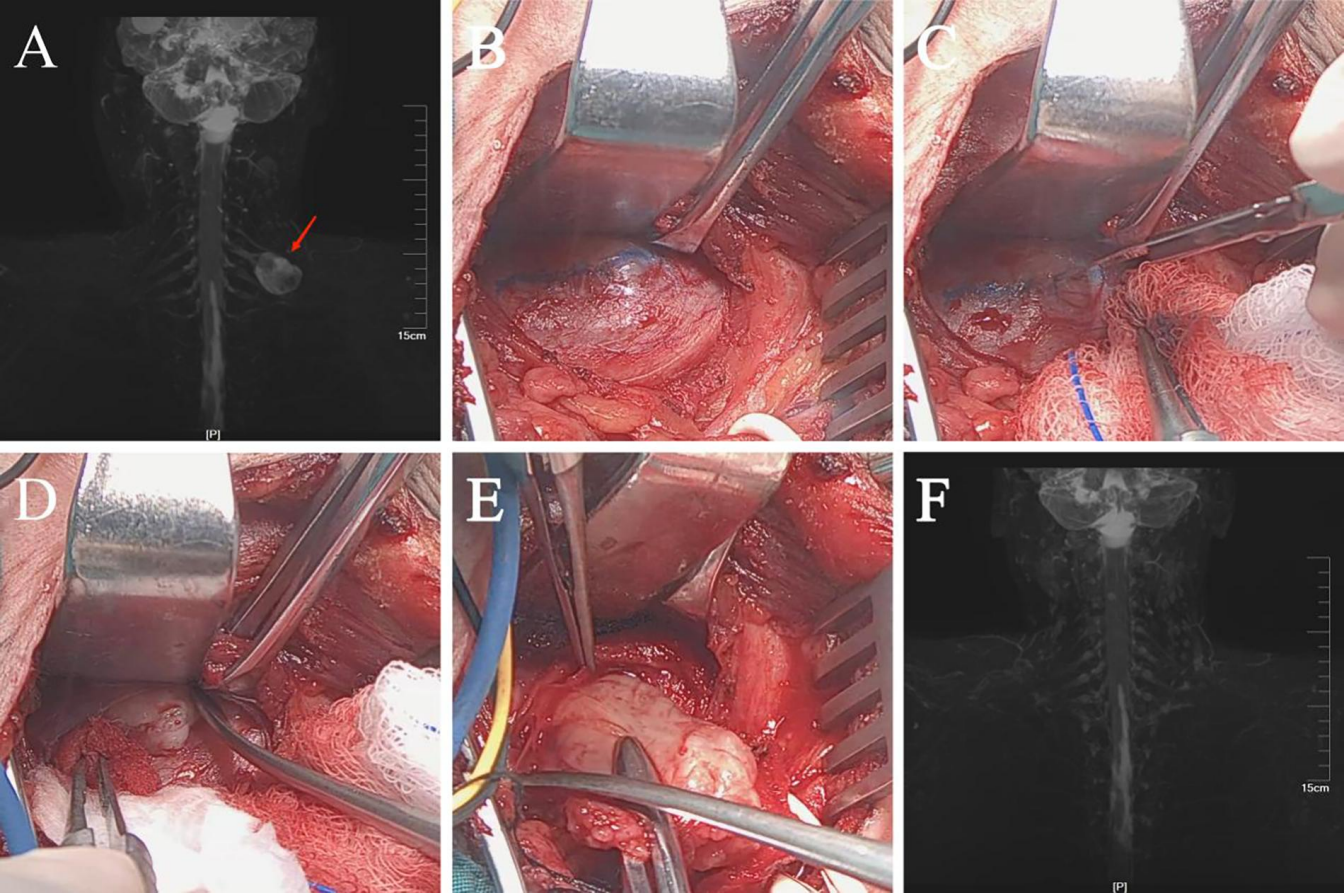
Brachial plexus schwannoma. **(A)** Magnetic resonance imaging (MRI) shows a round mass approximately 3.2 cm × 2.4 cm in size (arrow) in the left supraclavicular fossa. **(B)** The probe clearly identified the neural course and marked the incision route. **(C–E)** Nerve tracts were separated, and the tumor was excised intact from within the capsule. **(F)** One-year postoperative follow-up showed no abnormal signal in the surgical area. The morphology of and the signal from the brachial plexus showed no abnormality as well.

**Figure 4:**
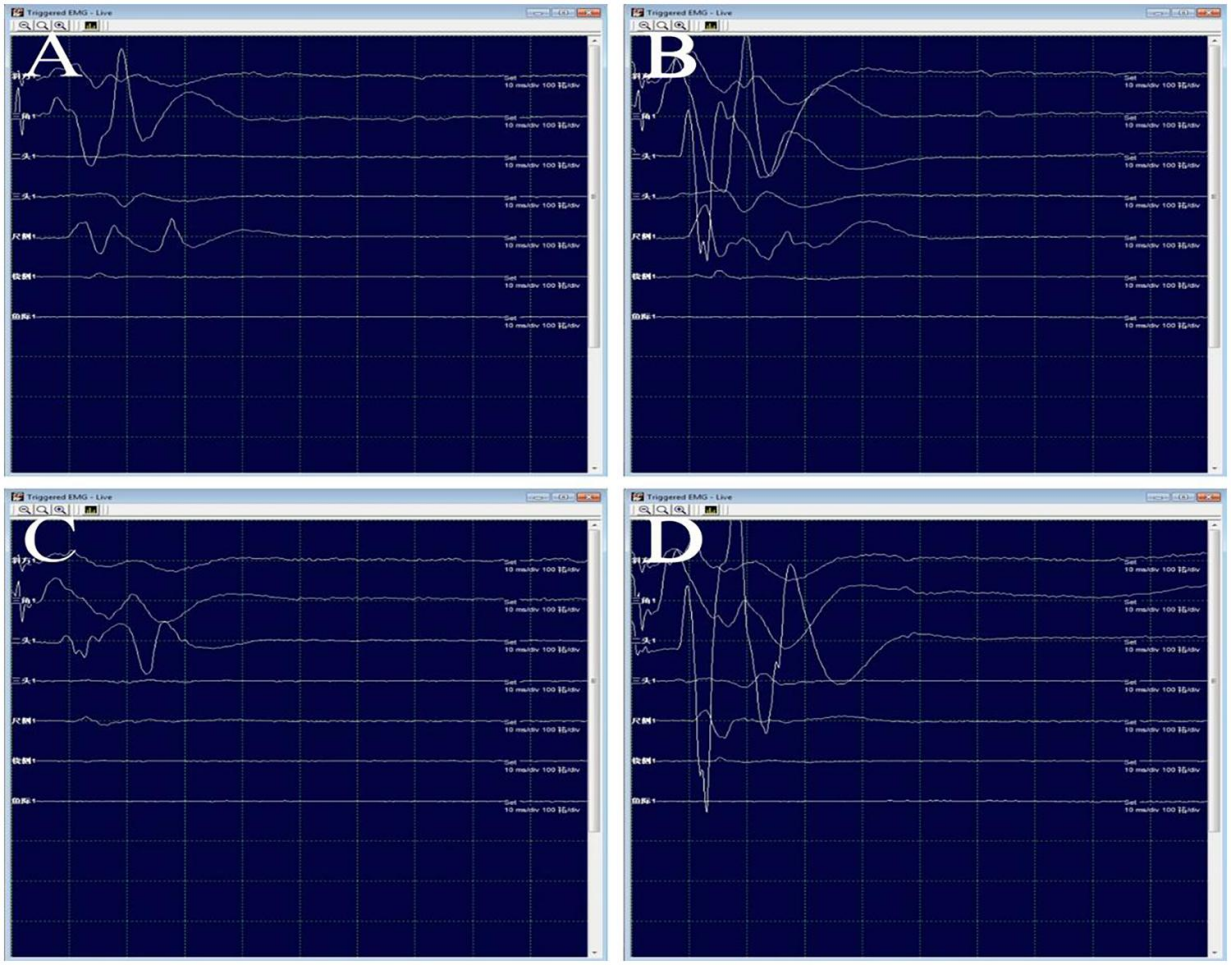
**(A–B)** During the operation, electrical stimulation of the C5 (a) and C6 (B) branches was performed with a probe. **(C–D)** After complete intracapsular resection of the tumor, the motor evoked potentials and stimulated electromyography were confirmed to be intact.

The procedure was performed with the assistance of IONM and high-definition endoscope. All patients were administered short-acting muscle relaxants under general anesthesia. A transverse incision, slightly longer than the maximium diameter of the tumor, was made according to the tumor location. Electrical stimulation along the direction of the nerve fibers on the tumor capsule was used to identify and mark the nerve bundles. When the clear tumor capsule was identified, the incision route was marked; the capsule was incised and peeled off gradually, the nerve bundle was carefully separated and finally led to the exposure of the tumor itself. We then gently lifted the tumor with non-destructive forceps to expose the tail of the tumor. The high-definition endoscope was applied at this point to approach and observe the pathway of nerve bundles on the inner surface of the capsule. Finally, the tumor was resected intact from within the capsule without any interference with surrounding nerve bundles. The stimulation probe was used again on both the inner and outer side of the capsule to make sure signals remained intact. The surgical cavity was then re-checked to ensure that neither residual neoplasm nor active bleeding was present. The fascia and skin were sutured in layers, and a negative-pressure drainage tube was inserted. The incision was covered with dressing. The patient was discharged on the second day after the removal of drainage the tube (Figures 2–4).

### 2.5 Follow-up

The patients were followed-up monthly and any postoperative neurological dysfunction were recorded. The patients were scanned with MRI for tumor recurrence 6 months after the surgery.

## 3 Clinical outcomes

 Table 1 is a summary of patients' information. This cohort included nine men and eleven women, aged 15–74 years. All patients underwent the aforesaid surgical procedures to achieve complete tumor resection. Two patients developed postoperative numbness of their fingers, one patient developed postoperative numbness of their shoulder and the numbness had resolved in all affected patients after a one-month follow-up. None of the patients experienced any postoperative motor nerve dysfunction. The followed-up period was one to four years, and no tumor recurrence was observed. Postoperative pathological examination confirmed schwannoma in all twenty cases.

**Table 1:** Patients' general condition and operation condition.

No.	Affected side	Sex	Age	Preoperative symptoms	Neural origin	Muscles monitored	Tumor size (cm)	Tumor resection	Postoperative complications
1	Left	Female	29	Pain in the left hand, numbness upon pressing on the tumor	C6	Deltoid, biceps brachii, triceps brachii, and brachioradialis	1.8	Intracapsular total resection	None
2	Left	Male	48	Numbness upon pressing on the tumor	C5/6	Deltoid, biceps brachii, tricepsbrachii, and brachioradialis	3.2	Intracapsular total resection	None
3	Left	Female	59	Numbness in the fingers of the left hand	C6/7	Deltoid, biceps brachii, triceps brachii, and brachioradialis	4.6	Intracapsular total resection	Numb fingers
4	Left	Female	72	Numbness upon pressing on the tumor	C6/7	Deltoid, biceps brachii, triceps brachii, and brachioradialis	3.5	Intracapsular total resection	None
5	Left	Male	36	Tumor found	C6/7	Deltoid, biceps brachii, triceps brachii, and brachioradialis	2.6	Intracapsular total resection	None
6	Left	Female	72	Numbness upon pressing on the tumor	C6/7	Deltoid, biceps brachii, triceps brachii, and brachioradialis	6.3	Intracapsular total resection	Numb fingers
7	Left	Male	27	Numbness upon pressing on the tumor	C5/6	Deltoid, biceps brachii, triceps brachii, and brachioradialis	4.2	Intracapsular total resection	None
8	Right	Female	17	Numbness in the fingertips	C6/7	Deltoid, biceps brachii, triceps brachii, and brachioradialis	3	Intracapsular total resection	None
9	Left	Female	74	Numbness upon pressing on the tumor	C6/7	Deltoid, biceps brachii, triceps brachii, and brachioradialis	3.2	Intracapsular total resection	None
10	Left	Male	65	Tumor found	C6/7	Deltoid, biceps brachii, triceps brachii, and brachioradialis	5.9	Intracapsular total resection	Numbness in the left shoulder
11	Left	Female	15	Tumor found	C5/6	Deltoid, biceps brachii, tricepsbrachii, and brachioradialis	4.5	Intracapsular total resection	None
12	Right	Male	29	Tumor found	C6	Deltoid, biceps brachii, triceps brachii, and brachioradialis	6.1	Intracapsular total resection	None
13	Right	Female	30	Numbness upon pressing on the tumor	C6/7	Deltoid, biceps brachii, triceps brachii, and brachioradialis	4.4	Intracapsular total resection	None
14	Left	Male	57	Tumor found	C5/6	Deltoid, biceps brachii, triceps brachii, and brachioradialis	3	Intracapsular total resection	None
15	Left	Female	32	Tumor found	C5	Deltoid, biceps brachii, triceps brachii, and brachioradialis	4.1	Intracapsular total resection	None
16	Left	Female	36	Numbness upon pressing on the tumor	C5	Deltoid, biceps brachii, triceps brachii, and brachioradialis	2.8	Intracapsular total resection	None
17	Left	Male	61	Tumor found	C5	Deltoid, biceps brachii, triceps brachii, and brachioradialis	4.2	Intracapsular total resection	None
18	Left	Male	61	Numbness upon pressing on the tumor, shoulders lifting	C5/6	Deltoid, biceps brachii, triceps brachii, and brachioradialis	2.4	Intracapsular total resection	None
19	Right	Male	24	Tumor found	C5/6	Deltoid, biceps brachii, triceps brachii, and brachioradialis	2.5	Intracapsular total resection	None
20	Right	Female	26	Tumor found	C7/T1	Triceps brachii, palmaris longus, flexor carpi ulnaris and abductor pollicis brevis	2.9	Intracapsular total resection	None

## 4 Discussion

The brachial plexus is the primary neural network responsible for sensory and motor innervation of the upper limb. Its injury can lead to significant functional impairment, underscoring the importance of nerve preservation during surgery in this region. They mainly innervate the sensation and movements of the upper limbs, shoulders, back, and chest (8). In human production and living activities, the sensory and motor functions of upper limbs are important indicators to evaluate individuals' labor capacity and quality of life. As the primary nerve responsible for the sensory and motor functions of the upper limbs, brachial plexus damage can lead to severe sensory and motor dysfunction and thus significantly reduce one's quality of life (9).

Intraoperative neuromonitoring (IONM) technology was first used by surgeons to monitor facial nerve function in the 1960s. In 1966, Shedd et al. (10) applied it to thyroid surgery for the first time and successfully reduced the incidence of recurrent laryngeal nerve injury. Intraoperative use of IONM can clarify the tumor-to-nerve bundle relationship, particularly in the case of brachial plexus schwannomas, which has affected nerve bundles stretching over the outer surface of the tumor capsule (11, 12). During tumor resection, a probe with electric stimulation is used to identify and mark the nerve bundles, and to plan the incision route. Blunt dissection of the capsule and perineurium adequately expose the tumor (13, 14). Throughout the procedure, no pressure was applied to the nerve bundles, nor did any pull or rotation by surgical instruments. Furthermore, it is necessary to adjust the bipolar electrocoagulation output or to relax tissues under tension promptly based on real time IONM signals. Waiting until the waveform returned to normal can minimize nerve damage from anatomical traction and tumor dissection (3, 15). These technical improvements are likely the reason for the undamaged neural function of the patients.

Endoscopy offer more flexible angles and clearer local views, helping the surgeon to directly observe blind spots within narrow spaces and distinguish the lesion from the normal tissue (16). The high-definition endoscopic system helps to achieve the surgical goals in a minimally invasive manner, and at the same time maximizes the protection of the patient's neurological function (17). During the operation, the limitation of space and viewing angles makes it challenging to distinguish the nerve bundle on the inner side of the capsule and thus causes patient's motor dysfunction. For example, when the tumor was pulled upward, it was difficult to see the tail of the tumor directly, so we used the high-definition endoscope-assisted system to closely observe the nerve near the tumor tail. The system helps to achieve complete intracapsular resection without causing the brachial plexus dysfunction.

**Figure 5:**
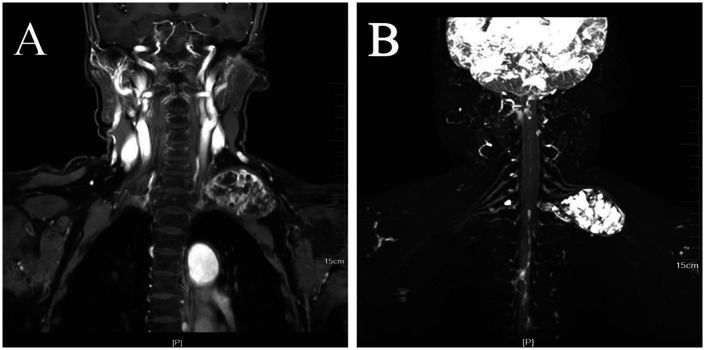
Images from different imaging sequences. **(A)** Conventional magnetic resonance imaging (MRI) sequence images. **(B)** 3D-STIR-SPACE sequence better exposed the tumor morphology.

The current imaging methods mainly include color Doppler ultrasound, CT, and MRI. They all can provide cross-sectional image of tissues and organs and MRI is the first choice for peripheral neuropathy evaluation. Clinicians use two-dimensional (2D) images (i.e., horizontal, coronal, and sagittal) to assess the location and size of the lesion but to determine its three-dimensional (3D) structure, the physician's clinical experience is required. Structures around the nerves tissues, such as blood vessels and lymph nodes, display similar signal and may influence the results (18). 3D-STIR-SPACE sequence provides reconstructions of multiplanar structures with maximum density to accurately decide the shape, size, and location of the neuropathy (19). It has important applications in diagnosing brachial plexus neuropathies (20), assuring adequate surgical plans and preventing intraoperative nerve and vascular injury. In our study, all patients underwent 3D imaging, which helped to better differentiate nerves from surrounding tissues and locate schwannomas more directly. This method facilitated the selection of surgical approaches and ensured the intraoperative protection of the nerves (Figure 5).

Lee et al. (21) conducted a retrospective analysis of 19 patients who underwent resection of brachial plexus tumors and found that 15% of patients experienced immediate postoperative sensory dysfunction, while 10% of patients experienced immediate postoperative motor dysfunction. Reexamination after three to five years of follow-up showed improvement of above symptoms. Huang et al. (22) analyzed 42 patients who underwent resection of brachial plexus tumors and found that 22% had postoperative neurological-related problems. Lee et al. (1) analyzed five patients who underwent brachial plexus surgery, and found that IONM led to a higher success rate of surgery and lower chance of complications. Ishida et al. (23) compared 103 patients who underwent spinal tumor resection with IONM and found that the sensitivity of IONM's overall pooled diagnostic value was 77.9%. Matsushima et al. (24) reviewed eighteen patients with neurofibromatosis and found that under adequate nerve monitoring, the tumor resection rates of 95%–100%. Unlike surgical approaches that involves removing the schwannomas together with its capsule, the use of IONM to plan the incision route allows for complete intracapsular resection of schwannomas and preserves the function of the majority nerve fibers. This is key to minimizing postoperative complications. Roh (25) studied schwannomas of different origins and concluded that intracapsular resection was the safe and effective treatment for brachial plexus schwannomas. We also believe that intracapsular resection with IONM and a high-definition endoscopic system is an easier and safer way than removing the tumor and its capsule altogether. In our study, all twenty patients underwent complete intracapsular resection, with two patients developed postoperative numbness of the fingers and one patient developed postoperative numbness of the shoulder. Their numbness disappeared after 1 month follow-up, and all the patients' postoperative motor function was unaffected. We confirmed that the usage of IONM is reliable, sensitive, and observable.

Successful application of IONM requires careful perioperative management. Key considerations include the maintenance of stable patient core temperature to prevent latency prolongation in evoked potentials, and a tailored anesthetic regimen (26). To avoid suppression of neuromuscular junction transmission and the consequent attenuation or loss of MEPs and EMG activity, the use of long-acting neuromuscular blocking agents is contraindicated after anesthesia induction (27). In this series, only short-acting neuromuscular blockers were administered during tracheal intubation, with anesthesia maintained using total intravenous anesthesia (TIVA) to ensure optimal neurophysiological signal quality throughout the procedure.

Although IONM has remarkable advantages in postoperative outcomes, most intraoperative neuromonitoring studies lack long-term results (28). Some studies suggested that unskillful use of IONM instruments might extend the operation time. Additionally, schwannoma in brachial plexus could be easily detected during the operation, leading some to believe that nerve monitoring is unnecessary. However, we believe that the protection of peripheral nerve function is more important than locating the tumor sheath alone during the operation. IONM plays an important role in locating and identifying the nerve pathways, recognizing any anatomic variations, making timely corrections to avoid intraoperative nerve injury and assessing the severity of nerve injury. The use of IONM increased the surgeon's sense of safety during surgery (29) and provided psychological comfort to patients and their families as they knew that the method to address potential nerve damage is always at hand (30).

In conclusion, the results of this series demonstrate that the combined use of intraoperative neuromonitoring and high-definition endoscopy facilitated complete intracapsular resection of brachial plexus schwannomas in all patients, with preservation of motor function and no recurrences at mid-term follow-up. This integrated approach provides the surgeon with enhanced visual precision and real-time functional feedback, which are critical for navigating complex neural anatomy. Our findings support the feasibility and safety of this technique as a valuable strategy for optimizing surgical outcomes while prioritizing nerve preservation in the management of these tumors.

Limitations: This study has several limitations. Firstly, it is a retrospective analysis with a relatively small sample size, which may limit the generalizability of the findings. Secondly, the absence of a control group precludes direct comparative efficacy analysis of the combined technique against conventional methods. Thirdly, postoperative neurological function was assessed in a descriptive manner without the use of standardized, validated functional scoring systems, which could provide a more objective and granular evaluation of outcomes.

## Data Availability

The original contributions presented in the study are included in the article/Supplementary Material, further inquiries can be directed to the corresponding authors.
